# Confrontation échographique et fœtopathologie après interruption thérapeutique de grossesse dans une maternité Tunisienne de référence

**DOI:** 10.11604/pamj.2016.25.256.10011

**Published:** 2016-12-21

**Authors:** Mehdi Kehila, Ahmed Halouani, Omar Touhami, Hassine Saber Abouda, Abdeljalil Khlifi, Rim Ben Hmid, Ines Benhassen, Aida Masmoudi, Mohamed Badis Chanoufi

**Affiliations:** 1 Service C du Centre de Maternité et de Néonatologie de Tunis, Faculté de Médecine de Tunis, Université Tunis El Manar, Tunisie; 2 Service de Gynécologie Obstétrique, CHU Farhat Hached, Faculté de Médecine de Sousse, Tunisie; 3 Service de Fœtopathologie du Centre de Maternité et de Néonatologie de Tunis, Faculté de Médecine de Tunis, Université Tunis El Manar, Tunisie; 4 Service de Radiologie, Hopital Habib Thameur, Université Tunis El Manar, Tunisie

**Keywords:** Fœtus, avortement, échographie-prénatale, malformation, autopsie, Fetus, interruption of pregnancy, prenatal ultrasound, malformation, autopsy

## Abstract

Nous proposons d’évaluer la pertinence du diagnostic échographique anténatal en le comparant aux conclusions de l’examen foetopathologique en cas d’interruption thérapeutique de la grossesse pour indication fœtale. Il s’agit d’une étude rétrospective descriptive et analytique menée sur une période de trois ans allant de janvier 2013 à décembre 2015 ayant porté sur 66 fœtus autopsiés à la suite d’une interruption thérapeutique de grossesse pour indication fœtale. L’examen fœtopathologie a confirmé les résultats de l'échographie dans 63 cas (95,4%). Dans 18 cas (27,2%) il y avait une concordance complète entre les résultats du diagnostic prénatal et ceux de l'autopsie. Neufs pour cent des malformations fœtales ont été détectées au premier trimestre. La majorité de malformations (72%) ont été détectées au deuxième trimestre. Les malformations neurologiques étaient les plus fréquentes (60%), dominées par l’hydrocéphalie et l’anencéphalie. Cette étude montre que, dans nos conditions d’exercice, même si le diagnostic échographique est souvent non exhaustif, les indications des interruptions thérapeutiques de grossesses sont correctes. L’examen foetopathologique vient dans ce cas compléter les malformations méconnues permettant de préciser le diagnostic et mettre en place une stratégie pour les grossesses ultérieures.

## Abstract (en)

This study aims to evaluate the value of prenatal ultrasound diagnosis by comparing it with the results of the fetopathological examination in case of therapeutic interruption of pregnancy for fetal indication. We conducted a retrospective descriptive and analytical study carried out over a three-year period from January 2013 to December 2015. It involved 66 fetuses autopsied after therapeutic interruption of pregnancy for fetal indication. Fetopathological examination confirmed ultrasound results in 63 cases (95.4%). In 18 cases (27.2%) there was a full match between the results of the prenatal diagnosis and those of the autopsy. Nine percent of fetal malformations were detected in the first trimester. The majority of malformations (72%) were detected in the second timester. Neurological malformations were the most frequent (60%), dominated by hydrocephalus and anencephaly. This study shows that, in our clinical context, even if ultrasound diagnosis is often non-exhaustive, its signs indicating the need for interruptions of pregnancy are correct. Fetopathological examination is used, in this case, to detect unknown malformations, making it possible to specify the diagnosis and to implement a strategy for subsequent pregnancies.

## Introduction

Les avancées techniques de l’imagerie fœtale ainsi que les avancées scientifiques ont bouleversé considérablement l’essor de la médecine fœtale, rendant l’immense majorité des malformations accessible à un diagnostic prénatal [1, 2]. La découverte d’une malformation à l’échographie anténatale est de ce fait devenue une situation fréquente [2]. Le risque majeur dans ces cas est d’indiquer une ITG pour un fœtus normal ou ne présentant pas de malformation majeure ou incurable. En effet, le diagnostic de certitude des anomalies congénitales suspectées ne sera établi que sur l’examen fœtopathologique [3–7]. Dans ce travail, nous nous sommes proposés d’évaluer la pertinence des diagnostics échographiques établis avant les ITG dans un centre universitaire Tunisien de référence et de calculer la valeur prédictive positive (VPP) de l’échographie dans le diagnostic des différentes malformations.

## Méthodes

Il s'agit d’une étude rétrospective descriptive et analytique à propos de tous les fœtus autopsiés au service de fœtopathologie du Centre de Maternité et de Néonatologie de Tunis (CMNT), à la suite d’une interruption thérapeutique de grossesse pour une indication fœtale réalisée au service "C" du CMNT entre le 1^er^ Janvier 2013 et le 31 Décembre 2015. Le CMNT est la maternité de référence de la capitale. Il s’agit d’un centre universitaire de niveau 3 réalisant une moyenne de 15 000 accouchements par an et drainant une grande partie des grossesses à haut risque du pays. Le service de foetopathologie du CMNT représente le seul service de foetopathologie de la capitale. Il draine aussi bien les fœtus du secteur public que privé et pratique en moyenne 800 autopsies par an. L’identification des cas a été faite à partir des registres du service de fœtopathologie. Le recueil des donnés a été fait à partir des dossiers du service de foetopathologie et ceux du service "C" du CMNT. Ont été inclus dans notre étude tous les examens fœtopathologiques réalisés dans le cadre d’une ITG provenant du service "C". Ont été exclus de notre étude toutes les interruptions médicales de grossesse pour indication maternelle ainsi que les dossiers dont les comptes rendus d’échographie n’étaient pas disponibles. Le protocole d'interruption thérapeutique de grossesse dans le centre de maternité est bien standardisé: lorsqu’une patiente est adressée pour une anomalie constatée à l’échographie ou lorsqu’un résident du service suspecte une anomalie à l’échographie anténatale, l’examen échographique est obligatoirement contrôlé par un senior du service pour confirmer l’anomalie. Dans les cas de doute, le dossier est staffé et l’échographie est refaite en staff en présence de tous les seniors de l’équipe. Après confirmation, l’anomalie est expliquée aux parents ainsi que ces conséquences et les différentes possibilités thérapeutiques existantes. Si les parents expriment une demande éclairée d’ITG, celle-ci doit être formulée par écrit et signée. Dans ce cas, le dossier est soumis à l'avis du comité d’éthique du CMNT. Les membres du comité d’éthique ont le droit de faire par eux même un contrôle échographique s'ils le jugent nécessaire. L'ITG ne pouvant être réalisée que suite à l'accord du comité d’éthique. L'ITG est réalisée en hospitalisation par du Misoprostol selon les protocoles recommandés par la FIGO en 2012 (http://www.figo.org/sites/default/files/uploads/project-publications/Miso/Misoprostol_Recommended%20Dosages%202012.pdf). Après expulsion, le fœtus et le placenta sont acheminés à l'état frais au service de fœtopathologie. L’examen fœtopathologique comprend systématiquement des photographies, des radiographies du fœtus ainsi qu’une autopsie. Pour chaque fœtus, nous avons comparé les données échographiques aux données de l’examen fœtopathologique. L’examen fœtopathologique a été considéré comme référence pour mesurer la valeur prédictive positive de l’échographie pour chaque malformation majeure diagnostiquée. Pour comparer les constatations échographiques sur lesquelles les indications des ITG ont été posées aux résultats des examens fœtopathologies, nous avons utilisé la classification proposée par Brand et al. [1]. Ainsi, selon le degré de concordance de la confrontation anatomo-échographique, 4 groupes ont été déterminés:

**GROUPE A:** Concordance totale entre le diagnostic échographique anténatal et l’examen fœtopathologique.

**GROUPE B:** L’examen fœtopathologique a confirmé le diagnostic échographique et y a ajouté d’autres malformations non diagnostiquées échographiquement.

**GROUPE C:** L’examen fœtopathologique n’a pas retrouvé les anomalies échographiques, mais il a diagnostiqué d’autres malformations au moins aussi grave que les constatations échographiques.

**GROUPE D:** Malformation(s) mineure(s) n’indiquant pas une ITG ou aucune malformation retrouvée à l’examen fœtopathologique. L’étude statistique a été réalisée par le logiciel XLSTAT (2014.4.09; Addinsoft, USA). La valeur prédictive positive de l’examen échographique a été calculée pour les malformations majeures retrouvées.

## Résultats

Soixante six cas d’interruption thérapeutique de grossesse (ITG) pour indication fœtale ont été colligés pendant la période d’étude. Deux ITG ont été réalisées au premier trimestre (3%), 52 au deuxième trimestre (78%), et 12 au troisième trimestre (19%). L’âge maternel moyen était de 33 ans (extrêmes 21 - 42). La gestité moyenne était de 3 (extrêmes 1 - 6). La parité moyenne était de 2 (extrêmes 0 - 6). Une consanguinité a été retrouvée dans 22,7% des cas. Un antécédent de fausse couche spontanée a été trouvé chez 18% des patientes. Dix-huit patientes ont eu une amniocentèse avant l’ITG. Aucune biopsie du trophoblaste n’a été réalisée. Une échographie du premier trimestre avec mesure de la clarté nucale a été pratiquée chez 45% des patientes. La malformation à l’origine de l’indication de l’ITG a été diagnostiquée à l’échographie du premier trimestre dans 4.5% des cas, à l’échographie du deuxième trimestre dans 77, 2% des cas et à l’échographie du troisième trimestre dans 18,3% des cas. Sur les 66 ITG réalisées, 18(27%) ont été classées dans le groupe A, 45 (68%) ont été classées dans le groupe B, 3(5%) ont été classées dans le groupe C et aucune ITG n’a été classée dans le groupe D. Les malformations neurologiques étaient les plus fréquentes, retrouvées dans 68% des cas. Le Tableau 1 résume le pourcentage des malformations retrouvées à l’échographie et à l’examen foetopathologique pour chaque système. Les valeurs prédictives positives de l’échographie pour les malformations majeures (ayant motivé l’ITG) sont résumées dans le Tableau 2. Le taux de faux positifs dans notre série était de 4,5%, ce taux correspond au groupe C de l’étude. En fait, ce groupe est formé par des fœtus chez qui la constatation échographique majeure ayant motivé l’ITG n’a pas été retrouvée à l’examen foetopathologique. Ceci ne signifie pas, dans ces cas, l’absence d’anomalie mais il s’agissait en fait d’une erreur de classification de l’anomalie constatée. Nous citons à titre d’exemple une meningo-encéphalocèle occipitale diagnostiquée à l’échographie comme anencéphalie.

**Tableau 1:** Pourcentage des malformations retrouvées à l’échographie et à l’examen fœtopathologie pour chaque système

Les malformations par système	Vues à l’échographie	Examen fœtopathologique
Malformations neurologiques	68%	68%
Malformations cardiaques	27%	36%
Malformations digestives	14%	22,7%
Malformations néphro-urologiques	18%	32%
Malformations du squelette et des extrémités	18%	59%
Anomalies de la face	14%	50%

**Tableau 2:** Valeur prédictive positive de l’échographie selon la malformation

Les malformations par système	VPP
Malformations neurologiques	73,3%
Malformations cardiaques	100%
Malformations digestives	100%
Malformations néphro- urologique	100%
Malformations du squelette et des extrémités	100%

## Discussion

L’examen échographique est devenu un examen indispensable dans le suivi anténatal. Il permet entre autres de faire le bilan morphologique du fœtus afin de diagnostiquer une éventuelle malformation. Quand une malformation majeure est découverte, 80% des couples décident d’interrompre la grossesse [8]. Il existe, néanmoins, deux problèmes majeurs avec ce diagnostic échographique anténatal: premièrement, les faux négatifs, qui signifient que des anomalies réellement existantes ne sont pas détectées à l’échographie anténatale; deuxièmement, les faux positifs, signifiants que les anomalies diagnostiquées à l’échographie anténatale ne sont pas retrouvées à la naissance ou à l’examen fœtopathologique après une ITG. La hantise étant de faire un ITG pour un fœtus sain. Ces deux situations ont des effets psychologiques négatifs lourds pour le couple et représentent même un problème médico-légal pour l’équipe soignante. Le taux de faux négatifs peut être diminué par un renforcement de la formation des dépisteurs de première ligne alors que pour diminuer le taux de faux positifs il faut travailler dans le sens de la sous spécialisation et avoir de bons échographistes référents capables de redresser le diagnostic avant une éventuelle ITG. Le fait aussi de revérifier à plusieurs niveaux (augmenter les filtres), comme ce qui est fait dans notre centre permet aussi de diminuer fortement ces faux positifs. En effet, ce système de filtres dans notre série nous a permis d’obtenir un taux de faux positifs de 0%. En analysant les différents groupes A, B, C et D, nous pouvons dire que le Groupe A peut être considéré comme techniquement complet et les groupes A, B et C comme cliniquement corrects vu que l’indication de l’ITG y était. Ainsi, nous n’avons été techniquement complets que dans 27% des cas. Cependant, nous avons été cliniquement corrects dans 100% des cas. Dans 63 cas (95%) les résultats de l'échographie étaient confirmés par l’examen fœtopathologique (groupe A + groupe B).

Dans notre série, Le taux de concordance totale était de 27%. Il s’agit d’un taux relativement faible comparé aux taux publiés dans la littérature, essentiellement par des équipes européennes, et qui vont de 46,8 à 88,1% (Tableau 3). L’examen fœtopathologique a confirmé le diagnostic échographique et y a apporté des éléments complémentaires (groupe B) dans 68% des cas contre 24 à 38,9% dans la littérature [9–15]. Cette différence peut entre autre être expliquée par la charge de travail très élevée dans notre centre, ne permettant pas de faire des examens échographiques exhaustifs. En effet, le temps alloué à chaque examen échographique ne dépasse pas 15 min en moyenne. Cette durée demeure insuffisante pour une étude morphologique détaillée. Il est vrai que dans notre structure nous disposons d’une unité d’échographie et de diagnostic anténatal, cependant, il n’y a qu’un seul médecin dédié à cette fin. L’autre raison est qu’une fois le diagnostic d’ITG pour une malformation fœtale majeure est posé par un échographiste, ce dernier ne s’attarde pas à chercher d’autres malformations. Cette constatation est par ailleurs retrouvée dans la série de Ramalho et al. [11]. Il est important, de voir le taux de patientes du groupe B diminuer au profit du groupe A. En effet, ceci nous permettra d’émettre un diagnostique anténatal plus pointu, et ainsi une meilleure information des parents. Ajouté à cela, un groupement syndromique complet peut mieux orienter le diagnostic afin de demander d’autres examens anténatals, qui ne seraient pas possibles après ITG, comme le caryotype ou les dosages enzymatiques sur liquide amniotique. Toutefois, nous notons dans notre série que dans tous les cas, les indications des ITG y étaient. L’examen fœtopatologique est venu par la suite donner un diagnostic exhaustif des malformations. Notre taux de faux positifs était de 4,5%. Ce taux est comparable aux taux de la littérature allant de 2,1 à 9,9 (Tableau 3). Cependant en aucun cas nous n’avons indiqué une ITG pour un fœtus sain. En effet tous les faux positifs étaient dans le groupe C, chez qui l’examen fœtopatologique a permis de préciser le type exact de la malformation vue à l’échographie. La première cause d’interruptions de grossesse dans notre série était les malformations neurologiques (68% des cas), ce qui concorde avec d’autres séries publiées dans la littérature [16, 17] où les principales malformations fœtales en cause d’interruption médicale de la grossesse étaient les malformations neurologiques et les multiples associations malformatives. Dans la série de Aslan H et al. [18], mené sur 463 ITG en Turquie, les malformations neurologiques ont représenté 57.6% des indications des ITG avec comme anomalies prédominantes l’anencéphalie et la spina bifida. Dans notre série, le taux de malformations cardiaques était de 27%. Ceci est dans le même ordre de grandeur de ce qui est déjà rapporté dans une série française publiée par Senat MV et al. en 2002 [19]. Notre étude monte bien que l’échographie a une très bonne valeur prédictive positive pour le diagnostic des malformations majeures. En effet, cette VPP était de 73,3% pour les malformations neurologiques majeures et de 100% pour tous les autres systèmes. De plus, même dans le cas des malformations neurologiques majeures, le pourcentage de 73,3% n’est pas expliqué par l’absence de toute malformation neurologique mais plutôt à un mauvais classement de la malformation neurologique objectivée. Ceci peut être expliqué par le fait qu’une malformation majeure (pouvant indiquer à elle seule une ITG) est généralement difficile à rater à l’échographie. En fin, une autopsie du fœtus réalisée par un pathologiste compétant est nécessaire pour fournir une information exhaustive des anomalies fœtales. Cette information est d’une importance capitale pour la prise en charge des grossesses ultérieures et encore plus dans nos conditions de pratique où le diagnostic échographique est souvent incomplet. De plus, l’examen fœtopathologique va permettre à l’échographiste ainsi qu’aux autres membres de l'équipe de médecine fœtale d’obtenir un feedback, qui est d'une très grande importance pour assurer le contrôle-qualité des examens échographiques réalisés.

**Tableau 3:** Comparaison de nos résultats aux autres études publiées dans la littérature [9–15]

	Nombre de cas	Concordance complète (groupe A)	Concordance majeure (groupe B)	Faux positif
Johns et al, 2004	47	46.8%	24 (51.1%)	2.1%
Ramalho et al, 2006	76	61%	38.9%	2.6%
Ceylaner et al, 2006	37	78.4%	–	–
Kaasen et al, 2006	228	58.4%	31.4%	9.9%
Akgum et al, 2007	107	77%	–	3.2%
Phadke SR et al, 2010	91	72.5%	25.3%	2.2%
Struksnaes C et al, 2015	1029	88.1%	9,7%	_
**Notre etude**	**66**	**27%**	**68%**	**4.5%**

## Conclusion

Dans les pays où manque l’hyperspécialisation et où les centres de référence sont souvent surchargés, le diagnostic échographique des malformations est souvent non exhaustif. Notre série montre que dans ces conditions de travail et avec les moyens humains disponibles nos diagnostics échographiques en cas d’ITG même si ils sont souvent incomplets, ils sont cliniquement corrects. La fiabilité de ces examens peut entre autres être expliquée par la multiplication des filtres et la coopération avec les différents membres du comité d´éthique qui pour la plupart revérifient les anomalies diagnostiquées diminuant ainsi les risques d´erreurs. Dans le cadre des ITG pour malformations fœtales, l´échographie prénatale est fiable lorsqu’elle met en évidence une malformation majeure. L’échographie et l´examen fœtopathologique doivent être considérés comme complémentaires. L’examen fœtopathologique est d’une importance capitale. Il vient compléter les malformations méconnues permettant de préciser le diagnostic et permettant la mise en place d’une stratégie pour les grossesses ultérieures.

### Etat des connaissances actuelles sur le sujet

- Dans les pays développés, ou en dispose d’une sous spécialisation en médecine fœtale, le diagnostic anténatal est très performant;
- Ainsi, le taux de concordance complète entre échographie anténatale et examen foetoathologique peut atteindre les 90%.

### Contribution de notre étude à la connaissance

- Le taux de concordance complète est plus faible dans les pays où la sous spécialité n’est pas encore au point;
- Toutefois, le diagnostic anténatal peut être fiable moyennant certaines précautions;
- L’examen fœtopathologie est capital dans ce contexte pour avoir un diagnostic précis et complet.
